# Rapamycin Improves Spatial Learning Deficits, Vulnerability to Alcohol Addiction and Altered Expression of the GluN2B Subunit of the NMDA Receptor in Adult Rats Exposed to Ethanol during the Neonatal Period

**DOI:** 10.3390/biom11050650

**Published:** 2021-04-28

**Authors:** Malgorzata Lopatynska-Mazurek, Anna Antolak, Pawel Grochecki, Ewa Gibula-Tarlowska, Anna Bodzon-Kulakowska, Joanna Listos, Ewa Kedzierska, Piotr Suder, Jerzy Silberring, Jolanta H. Kotlinska

**Affiliations:** 1 Department of Pharmacology and Pharmacodynamics, Medical University, Chodzki 4A, 20-093 Lublin, Poland; gosia.lopatynska@gmail.com (M.L.-M.); pawel.grochecki@umlub.pl (P.G.); ewa.gibula@umlub.pl (E.G.-T.); joanna.listos@umlub.pl (J.L.); ewa.kedzierska@umlub.pl (E.K.); 2 Department of Analytical Chemistry and Biochemistry, Faculty of Materials Sciences and Ceramics, AGH University of Science and Technology, Mickiewicza 30 Ave., 30-059 Krakow, Poland; antolak@agh.edu.pl (A.A.); abk@agh.edu.pl (A.B.-K.); piotr.suder@agh.edu.pl (P.S.); jerzy.silberring@agh.edu.pl (J.S.)

**Keywords:** rapamycin 1, spatial learning 2, reward 3, neonatal ethanol exposure 4, adult rats 5

## Abstract

Ethanol exposure during pregnancy alters the mammalian target of rapamycin (mTOR) signaling pathway in the fetal brain. Hence, in adult rats exposed to ethanol during the neonatal period, we investigated the influence of rapamycin, an mTOR Complex 1 (mTORC1) inhibitor, on deficits in spatial memory and reversal learning in the Barnes maze task, as well as the ethanol-induced rewarding effects (1.0 or 1.5 g/kg) using the conditioning place preference (CPP) paradigm. Rapamycin (3 and 10 mg/kg) was given before intragastric ethanol (5 g/kg/day) administration at postnatal day (PND)4–9 (an equivalent to the third trimester of human pregnancy). Spatial memory/reversal learning and rewarding ethanol effect were evaluated in adult (PND60–70) rats. Additionally, the impact of rapamycin pre-treatment on the expression of the GluN2B subunit of NMDA receptor in the brain was assessed in adult rats. Our results show that neonatal ethanol exposure induced deficits in spatial memory and reversal learning in adulthood, but the reversal learning outcome may have been due to spatial learning impairments rather than cognitive flexibility impairments. Furthermore, in adulthood the ethanol treated rats were also more sensitive to the rewarding effect of ethanol than the control group. Rapamycin prevented the neonatal effect of ethanol and normalized the GluN2B down-regulation in the hippocampus and the prefrontal cortex, as well as normalized this subunit’s up-regulation in the striatum of adult rats. Our results suggest that rapamycin and related drugs may hold promise as a preventive therapy for fetal alcohol spectrum disorders.

## 1. Introduction

Alcohol consumption during pregnancy has profound effects on structure and function in the developing human brain. Fetal alcohol spectrum disorder (FASD) is a term used to describe the effects that can occur in an individual with prenatal alcohol exposure [1]. These effects can include cognitive, emotional and motor deficits, together with characteristic morphological abnormalities [2,3]. In addition to verbal working learning disabilities, children with FASD struggle with spatial memory deficits that are directly related to abnormalities in the development of the hippocampus [4]. Indeed, spatial memory has emerged as a dominant deficit in individuals with FASD, that is consistent in children, adolescents and adults [5].

Previous study demonstrated mechanistic similarities between spatial learning and memory and the rewarding effects of addictive drugs, including ethanol [6,7]. The brain regions and neural processes that underlie addiction overlap extensively with those that support cognitive functions, such as learning, memory and abstract reasoning. Drug activity in these regions and processes during early stages of abuse foster strong maladaptive associations between drug use and environmental stimuli that may underlie future cravings and drug-seeking behaviors [8,9]. Published data shows that FASD can be a risk factor for the use and abuse of drugs in later life and various authors report that in animal studies, perinatal alcohol exposure increases alcohol preference, intake [10,11] and alcohol-induced reward later in life [12,13,14]

N-methyl-D-aspartate (NMDA) is an ionotropic glutamatergic receptor important during brain development, synaptic plasticity and learning and memory processes. NMDA receptor dependent long-term potentiation (LTP) and long-term depression (LTD) are signal transmission forms of neural circuits taking part in learning and memory and drug dependence [15,16,17]. Ethanol exposure during the fetal period of life disrupts LTP and LTD—cellar mechanisms for learning and memory [18]. Ethanol is a NMDA antagonist and ethanol exposure during pregnancy alters the NMDA subunits expression in the adult brain. The GluN2B subunit of NMDA is very sensitive to alcohol exposure, in particular. It has been previously reported that prenatal ethanol exposure reduces the GluN2B expression in both juvenile and adult hippocampus [19], particularly in male individuals [20].

The mammalian target of rapamycin (mTOR), a serine-threonine kinase, is associated with two distinct multi-protein signaling complexes, mTORC1 and mTORC2. mTOR signaling pathways occur in brain structures associated with memory and cognitive functions, including the hippocampus, amygdala, nucleus accumbens and prefrontal cortex [21,22]. mTORC1, of which Raptor is an essential component, plays a central role in cell growth by regulating protein synthesis and turnover, in addition to lipid, nucleotide and glucose metabolism [23]. In the central nervous system (CNS), mTORC1 is a mediator of synaptic plasticity [24]. Rapamycin (sirolimus) is a naturally occurring macrolide that potently inhibits mTORC1 activity by preventing mTOR-protein complex formation [25,26]. Published data have shown that rapamycin reduces the neurotoxic effects of ethanol by inhibiting mTORC1, thus, it is neuroprotective [27,28].

The primary aim of our research is to examine whether the mTORC1 inhibitor, rapamycin, when used as pre-treatment before every ethanol administration during the neonatal period, can protect adult rats against the deleterious effect of ethanol on learning and memory. We employed the Barnes maze task to evaluate spatial memory. A secondary aim was to determine whether the rapamycin pre-treatment can similarly protect against enhanced rewarding effect of ethanol as measured by the conditioned place preference (CPP) test—in adult rats with prenatal ethanol exposure. Furthermore, we evaluated the influence of rapamycin on the downregulation of the GluN2B subunit of the NMDA receptor (Western blot assay) in adult rats in the prefrontal cortex and hippocampus. Such brain structures are involved in learning and memory and the rewarding effects of ethanol [29,30]. To perform the aforementioned experiments, ethanol was given to rat pups (male and female) over postnatal days (PND)4–9. This period is equivalent to the third trimester of human pregnancy [31,32].

## 2. Materials and Methods

### 2.1. Animals

**Figure 1:**
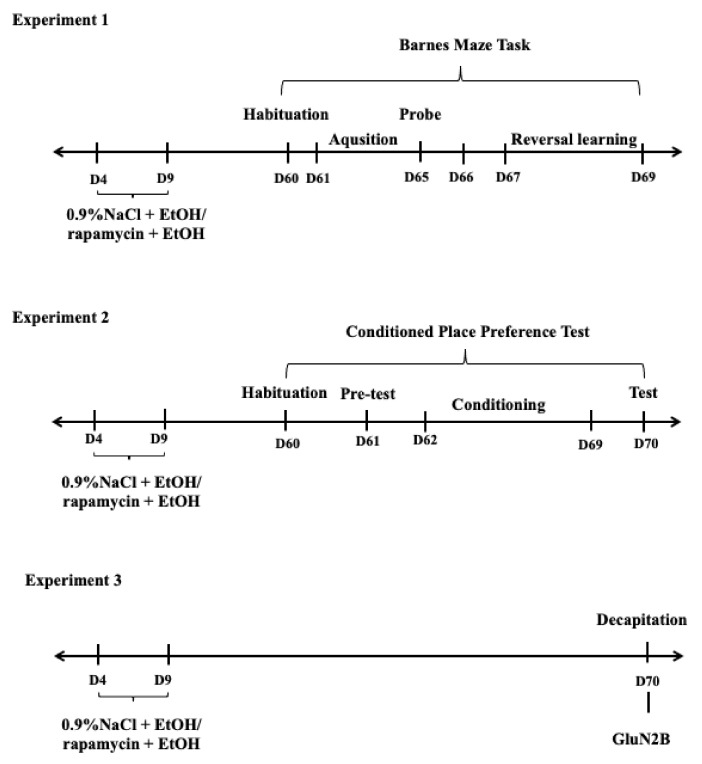
The diagram of experimental design. D-Days, EtOH-ethanol.

The outbred Wistar rats were housed in the cages (55 cm × 33 cm × 20 cm) in the vivarium of the Medical University of Lublin under standard laboratory conditions: constant and controlled temperature (22 ± 1 °C) and humidity (55 ± 10%), natural light/dark cycle (12 h/12 h) with lights on at 8:00 AM and free access to standard laboratory chow (Sniff Spezialdiäten GmbH, Soest, Germany) and water ad libitum. Firstly, one male and one female rat were housed together for one week for breeding and 3 weeks after mating, the females were checked each morning and evening for parturition. The day of birth was designated as postnatal day (PND) 0 and litters were culled to 8–10 pups on PND2. After this procedure on PND3, the pups were divided into double 6 (male and female) experimental groups (see below). (Experiment 1: learning and memory paradigm, Experiment 3: biochemical analysis) and 4 (male) experimental groups (Experiment 2: CPP paradigm), 8 pups each and paw-marked for identification. These rats were housed with dams to PND21. They were then separated into same-sex groups and housed until PND60 (Figure 1).

The experiments were carried out according to the National Institute of Health Guidelines for the Care and Use of Laboratory Animals, as well as to the European Community Council Directive for Care and Use of Laboratory Animals (86/609/EEC) and were approved by the Local Ethics Committee (137/2018).

### 2.2. Neonatal Ethanol Exposure and Rapamycin Administration

On PND3 male and female rats were paw-marked for identification and assigned into groups according to carried out experiments (Table 1). In the experiments, ethanol (95% *w*/*v*, Polmos, Poznan, Poland) was diluted with milk (Bebilon 1 Pronutra Plus, Nutricia, Warsaw, Poland) and administered intragastrically (i.g.), at the dose of 5.0 (22.66%, *v*/*v*) g/kg during PND4–9 according to the method described previously [33]. Rapamycin (Selleckchem, Munich, Germany) was dissolved in 0.9% NaCl and given intraperitoneally (i.p.), 1 h before ethanol/sham intubation at the dose of 3 or 10 mg/kg.

### 2.3. Barnes Maze Task

The Barnes maze task was conducted as published previously [34,35,36,37,38]. This test consisted of the following phases: adaptation phase (habituation), acquisition phase, test phase (probe trial) and reversal learning.

The Barnes maze apparatus is a round metal platform made of gray metal, 122 cm in diameter, placed 90 cm above the floor. On its circumference, holes are located every 20 cm (10 cm diameter). One of these holes is equipped with an escape box measuring 35 cm × 12 cm × 12 cm. The other holes are blocked. The escape box is both a shelter and a destination for the animals. Because all holes look identical, the experimental animals cannot discriminate the escape box hole from other holes until situated adjacent to it.

On the walls of the testing room, at a distance of 1–2 m from the surface of the platform, numerous visual cues (in the form of large, colorful geometric shapes) are arranged that allow the test animal to locate the escape box. To evoke escape response, the platform is brightly lit (500 W).

#### 2.3.1. Habituation Phase

This phase was intended to introduce the animals to new conditions and reduced anxiety behaviors. One day before the acquisition phase, the rats were placed on the center of the apparatus and allowed to freely explore the maze.

#### 2.3.2. Acquisition Phase

The acquisition phase included the process of teaching animals the way to the shelter. The experiment began 24 h after habituation phase and consisted of one training session per day for 5 consecutive days. Each training session was composed of 3 trials lasting 180 s. Each trail began with placing the rodent in the center of the platform. The animal was then left to explore the platform freely. The trials ended after 180 s or earlier, once the animal had entered the escape box. If the rat could not find the escape box within the time allowed, it was gently guided to it and also left inside the box for 30 s to gain acquaintance with it.

At the end of each test, the animals were returned to their home cages and the platform surface was washed with 10% (*v*/*v*) ethanol solution to eliminate olfactory impressions. During the acquisition phase, primary latency and primary error were scored. Primary latency is defined as the time to when the animal entered the escape box. Primary error is defined as the number of holes visited by the animal before discovering the escape box.

#### 2.3.3. Probe Trial

This test began 24 h after the end of the acquisition phase, on PND66. One 90 s session was carried out for each animal. During the probe trial, the escape box was removed, and all holes were blocked. Primary latency and primary error were counted. The probe trial has been used to assess the spatial memory of rodents.

#### 2.3.4. Reversal Learning

One day (24 h) after the probe trial, three sessions of three days duration of reversal learning trials were carried out (PND67–69). The conditions of this experiment were identical to those occurring during the acquisition phase (presence of an escape box, the platform being brightly lit), the location of the escape box, however, was positioned 180° differently. As a result, the animals were compelled to look for the escape box using the same spatial signals, while learning its new location. This experience was used to evaluate the flexibility of memory. In reversal learning, the primary latency and primary error were counted.

### 2.4. Preparation of Synaptosomal Membranes from NR2B Subunits of NMDA Receptor Expression

The procedure of sample preparations has been described elsewhere [39,40,41] and was used in our study with minor modification. To increase the concentration of NR2B subunit of NMDA receptors in the sample, synaptosomes were isolated from the prefrontal cortex and hippocampus on PND70. Unless otherwise stated, all reagents in this paragraph were taken from Sigma-Aldrich/Merck local supplier.

During the first step, tissue was homogenized using a mechanical homogenizer (BioGen PRO200, PRO Scientific, Oxford, CT, USA). Homogenization was performed in the volume ratio of 1 to 20 of original wet tissue weight (*w*/*v*). The cold buffer contained 0.32 M sucrose, 5 mM HEPES, 1 mM EDTA, 1 mM NaHCO_3_,1 mM MgCl_2_, as well as a protease inhibitor cocktail (Roche, Mannheim, Germany). The homogenate was then centrifuged for 10 min at 1000× *g*, at 4 °C. This step allowed for separating the cells’ nuclei. These were retained in a pellet. The obtained supernatant was subsequently centrifuged for 20 min at 20,000× *g*, at 4 °C using 5804R centrifuge (Eppendorf AG, Hamburg, Germany), to obtain a crude mitochondrial/synaptosomal pellet. Such pellet was contaminated with free mitochondria membrane fragments and myelin; hence, to remove those contaminants, it was resuspended once again in homogenization buffer and centrifuged for 20 min at 20,000× *g*.

The obtained crude membrane fraction was resuspended in hypotonic homogenization buffer (first buffer without sucrose) and homogenized for 30 s using( ultrasonic homogenizer, model UP100H (Hielscher Ultrasonics GmbH, Teltow, Germany ) and, finally, stirred on ice for 20 min. This step brings about the tearing of the membrane of synaptosomes due to the hypotonic character of the buffer. The obtained solution was subsequently centrifuged at 25,000× *g* at 4 °C for 20 min. The final pellet with synaptosomal membrane fraction was then resuspended in hypotonic homogenization buffer. After final centrifugation, 25,000× *g* at 4 °C for 20 min, samples were resuspended in hypotonic homogenization buffer supplemented with 4% of sodium dodecyl sulfate in order to enhance the release of proteins from the cell membrane. Exact protein concentration in samples was measured using the bicinchoninic acid method [42].

#### SDS-PAGE Electrophoresis and Western Blot Analysis

The 22 µL of sample was mixed with 22 µL of Laemmli sample buffer (65.8 mMTris-HCl, pH 6.8 26.3% (*w*/*v*) glycerol, 2.1% SDS, 0.01% bromophenol blue, with β-mercaptoethanol (final concentration 355 mM)), all reagents from BioRad company (Hercules, CA, USA) and heated for 10 min, at 99 °C. Then, 38 µL of such sample was placed onto 10% SDS-PAGE gel according to the Bio-Rad manufacturer’s protocol. Proteins were separated at 120 V for about 45 min.

Subsequently, to perform Western Blot analysis, the proteins were electro-transferred overnight onto Immun-Blot^®^ PVDF membrane (BioRad, Hercules, CA, USA) in TG (Tris/Glycine) buffer, at 4 °C, with an electric current of 100 mA. The blotted membrane was blocked with Casein Solution (Vector Laboratories, Burlingame, CA, USA) for 60 min at room temperature and washed three times with Tris-buffered saline (TBS) buffer (Sigma-Aldrich, Saint Louis, MS, USA). The membrane was then incubated with primary rabbit antibody: NMDAR2B Ab (Thermo Fisher Scientific, Rockford, MA, USA) in a 1:1000 dilution in TBS for 80 min at room temperature. The membrane was subsequently washed 3 × 10 min in Tris-buffered Saline with 1% Tween (TBST). After this, Goat anti-Rabbit IgG (H + L) Secondary Antibody HRP conjugate (Thermo Fisher Scientific, Rockford, MA, USA) in a 1:3000 dilution in TBST was added for 80 min at room temperature. The membrane was washed 2 × 10 min in TBST and 1 × 10 min in TBS. The antibodies were then visualized by horseradish peroxidase (HRP)—incubation in 1 Step Ultra TMB Blotting Solution (Thermo Fisher Scientific, Rockford, MA, USA) and then washed multiple times in H_2_O_dd_.

After the whole procedure, blots were recorded using a Gel DOC-XR + gel documentation system, ver. 5.2 (BioRad, Hercules, CA, USA). For densitometric analyses, the bands were quantified using Image Lab^TM^ analysis software (BioRad, Hercules, CA, USA). During this task, NR2B subunit expression was calculated against the total protein amount in the sample, which was equalized based on the spectrophotometric measurement using the bicinchoninic acid methodology [42].

### 2.5. Conditioned Place Preference (CPP)

The apparatus for performing CPP test consisted of 6 identical rectangular boxes divided into two rooms with the same dimensions (65 cm × 35 cm × 30 cm), differing in color and texture of the floor. Between the rooms there was a square passage closed with a guillotine, which, when opened, allowed for free movement of animals between the two parts of the apparatus. The walls and floors of the two large compartments differed in color and pattern. One was black and the other was with vertical black and white stripes. The testing room’s luminance was adjusted so that the environmental (visual and tactile) cues did not produce a significant baseline preference for a specific chamber. Between each test procedure, the whole apparatus was cleaned with 10% ethanol solution to neutralize the odor trail. The boxes were kept in a soundproof room with neutral noise masking and dim 40 lx illumination. The CPP performance was measured by computerized video tracking. The data collected in the CPP test were expressed as means ± SEM of preference scores (i.e., the differences between post-conditioning and pre-conditioning time spent in the drug-paired compartment).

#### CPP Procedure

The CPP paradigm was carried out according to the method described earlier [43] with minor modifications. The test consisted of several phases ongoing for 11 consecutive days: habituation (1 day), pre-test (1 day), conditioning (8 days) and test (1 day), as described below.

This experiment was conducted on adult male rats and started on PND60. The amount of time spent in each compartment was measured during the pre-test phase. These results were employed to separate animals into groups with approximately equal biases for each side. Moreover, an appropriate control group (0.9% NaCl-treated during all phases of experiments) was used that had undergone the same CPP procedure as the drug-treated rats.

On the first day of testing (habituation phase, PND60), all rats were placed in the central gray square compartment of the apparatus for 15 min and had free access to both rooms and were allowed to freely explore. No drugs were administered.

During the second day (pre-test, PND61), the procedure was the same as the habituation phase, but computerized video tracking was performed in order to measure the time the rats were present in particular parts of the apparatus. The analysis showed no preferences for any of the rooms.

During the third phase (conditioning, PND62–69), the animals were separated into 2 groups and conditioned twice daily (morning and evening shift) for eight consecutive days with at least a 3 h rest period between conditionings. In this phase, the animals were given ethanol (11.33% *v*/*v*, 1.0 or 1.5 g/kg) or the same volume of 0.9% NaCl. The rats were injected immediately before starting the experiment and were then placed for 30 min into the conditioning chamber with the guillotine doors separating the central gray area from the two compartments closed. The volume of each i.p. injection (1 mL/kg) was related to the weight of the rat.

During the last phase (test, PND70), the rats were placed individually in the CPP apparatus and left for 15 min with free access both compartments. The animals were not injected during this phase. The amount of time spent by animals in each compartment was then measured and recorded. All phases were conducted under the same conditions and the same techniques were applied. During the test phase, locomotor activity was measured for 15 min. Locomotion was measured as a total distance travelled (m) during the session.

### 2.6. Statistical Analyses

The obtained results were analyzed using Prism v. 8.0.0 for Windows (GraphPad Software, San Diego, CA, USA) and R v. 3.6.0. for Windows (four-way ANOVA). The statistical significance of drug effects from behavioral and biochemical tests was analyzed by the two-way or three-way analysis of variance (ANOVA) with repeated measures. This was followed by Bonferroni’s post-hoc test. The results were presented as means ± standard errors of means (SEM) of values. *p* value less than 0.05 was considered statistically significant for all tests.

## 3. Results

### 3.1. The Influence of Rapamycin Pre-Treatment before Every Ethanol Administration during PND4-9 on the Acquisition Memory of the Barnes-Maze Task in Adult (PND61–65) Male and Female Rats

Acquisition of spatial memory in the training phase was evaluated by a decrease in the latency time and the number of errors to reach the escape box for five consecutive days (PND61–65). A four-way ANOVA with repeated measures showed a significant effect of rapamycin pre-treatment (F(2,464) = 43.22; *p* < 0.001), ethanol treatment (F(1,464) = 51.11; *p* < 0.001), testing day (F(4,464) = 485.6; *p* < 0.001) and no significant effect of sex (F(1,464) = 0.3471; *p* > 0.05). Analysis also showed significant interaction between sex x testing day (F(4,464) = 3.577; *p* < 0.01) and no interactions between sex x ethanol (F(1,464) = 0.2342; *p* > 005) and sex x rapamycin pre-treatment (F(2,464) = 1.273); *p* > 0.05) in male adult rats (PND61–65).

In the number of primary errors committed by rats, a four-way ANOVA with repeated measures showed a significant effect of rapamycin pre-treatment (F(2,464) = 50.17; *p* < 0.001), ethanol administration (F(1,464) = 72.17; *p* < 0.001), testing day (F(4,464) = 309.1; *p* < 0.001) and significant contribution of sex factor (F(1,464) = 143.4 *p* < 0.001). Analysis also showed significant interaction between sex x testing day (F(4,464) = 68.13; *p* < 0.001), sex x ethanol (F(1,464) = 4.488; *p* < 0.05) and no significant interactions between sex x rapamycin pre-treatment (F(2,464) = 0.6801); *p* > 0.05) in adult rats (PND61–65).

Four-way ANOVA indicated that there is a main effect of sex interaction with other factors; therefore, separate analysis of the male and female data was performed.

**Figure 2:**
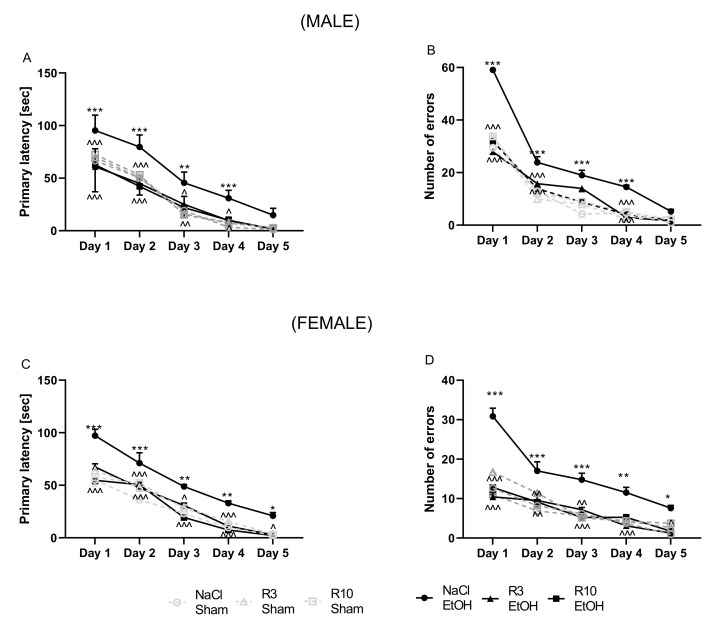
The influence of rapamycin pre-treatment (3 and 10 mg/kg, i.p.) during neonatal period before every ethanol administration (PND4–9) on the acquisition learning impairments in adult (PND61–65) male and female rats in the Barnes maze task. Primary latency (**A**,**C**) and number of errors (**B**,**D**) are expressed as mean ± SEM of 8 subjects per group (R 3.6.0. for Windows). * *p* < 0.05; ** *p* < 0.01 *** *p* < 0.001 vs. SI; ^ *p* < 0.05; ^^ *p* < 0.01; ^^^ *p* < 0.001 vs. EtOH. R3–rapamycin 3 mg/kg; R10–rapamycin 10 mg/kg, SI–shame intubation (i.g.); EtOH–ethanol intubation (i.g.).

Male: Acquisition of spatial memory in the training phase was evaluated by decrease the latency time and the number of errors to reach the escape box for five days (PND61–65). A two-way ANOVA with repeated measures showed a significant effect of day of acquisition learning (F(5,210) = 44.51; *p* < 0.001) and group of rats’ effect (F(4,210) = 433.7; *p* < 0.001) and interaction between these factors (F(20,210) = 1.888; *p* < 0.05) in male adult rats (PND61–65). Moreover, the post-hoc test demonstrated that latency time to reach the escape box was impaired following neonatal ethanol exposure on the 1st day (*p* < 0.001), 2nd (*p* < 0.001), 3rd (*p* < 0.001), 4th (*p* < 0.001) days but no on the 5th (*p* > 0.05) day of the trial. Pre-treatment with rapamycin (3 mg/kg, i.p.), decreased the primary latency on the 1st (*p* < 0.001), 2nd (*p* < 0.001), 3rd (*p* < 0.05), 4th (*p* < 0.05) days but no on the 5th (*p* > 0.05) day of acquisition learning trial. In male rats, pre-treatment with rapamycin (10 mg/kg) significantly decreased the primary latency to reach the escape box on the 1st (*p* < 0.001), 2nd (*p* < 0.001), 3rd (*p* < 0.01), 4th (*p* < 0.05) days but no on the 5th (*p* > 0.05) (Figure 2A). The results showed that there were no significant differences between neonatal ethanol and rapamycin groups as compared with the controls in the primary latency during the acquisition of the Barnes maze task (*p* > 0.05).

In the number of primary errors committed by rats, a two-way ANOVA with repeated measures indicated significant effect of day of acquisition learning (F (5,210) = 140.9; *p* < 0.001), group of rats (F(4,210) = 1094; *p* < 0.001) and interaction between these two factors in acquisition learning (F(20,210) = 18.79; *p* < 0.001) in male adult rats (PND61–65). The post-hoc test showed that the ethanol exposed group committed significantly more errors in finding the escape box than did the control group on the 1st (*p* < 0.001), 2nd (*p* < 0.001), 3rd (*p* < 0.01) and 4th (*p* < 0.05) days but no on the 5th (*p* > 0.05) day trials. Rapamycin pre-treatment at the dose of 3 mg/kg more significantly reversed this ethanol effect (on the 1st, 2nd and 4th day; *p* < 0.001) than at the dose of 10 mg/kg (on the 1st day (*p* < 0.001), 2nd day (*p* < 0.001) and 4th day (*p* < 0.001)). Rapamycin pre-treatment on both doses did not show this effect on the 3rd and 5th days (*p* < 0.05). (Figure 2B) in adult male rats (PND61–65). The results showed that there were no significant differences between neonatal ethanol and rapamycin groups as compared with the controls in the errors during the acquisition of the Barnes maze task (*p* > 0.05).

Female: Acquisition learning trials were performed the second day of experiment. A two-way ANOVA with repeated measures uncovered the significant effect of days of acquisition learning (F(5,210) = 53.13; *p* < 0.001), group of rats (F(2,210) = 359.0; *p* < 0.001) and interaction between groups x days (F(20,210) = 1.948; *p* < 0.05). In addition, the post-hoc test (Tuckey) showed that latency to reach the escape box was impaired following neonatal ethanol exposure (5 days (PND4–9) at the dose 5 g/kg, 22.66% *v*/*v*, i.g. on the 1st (*p* < 0.001), 2nd (*p* < 0.001), 3rd (*p* < 0.01), 4th (*p* < 0.01) and the 5th (*p* < 0.05) day of the trial. Pre-treatment with rapamycin (3 mg/kg, i.p.) equally reduced the primary latency on the 2nd (*p* < 0.001), 3rd (*p* < 0.05), 4th (*p* < 0.001) and the 5th (*p* < 0.05) day but did not do so on the 1st (*p* > 0.05) day of acquisition learning trials. In addition, pre-treatment with rapamycin (10 mg/kg) reduced the primary latency on the 1st (*p* < 0.001), 2nd (*p* < 0.001), 3rd (*p* < 0.001), 4th (*p* < 0.001) and the 5th (*p* < 0.05) days of acquisition learning in female adult rats (PND61–65) (Figure 2C). The results showed that there were no significant differences between neonatal ethanol and rapamycin groups as compared with the controls in the primary latency during the acquisition of the Barnes maze task (*p* > 0.05).

With regard to the number of errors committed by the female rats, a two-way ANOVA with repeated measures indicated the significant effect of days (F(5,210 = 57.32; *p* < 0.001), groups (F(4,210) = 106.2; *p* < 0.001) and groups x days interactions (F(20,210) = 4.201; *p* < 0.001) in the acquisition learning task. The post-hoc test (Tuckey) demonstrated that the ethanol exposed group committed much more errors in finding the escape box than did the control group on the on the 1st (*p* < 0.001), 2nd (*p* < 0.001), 3rd (*p* < 0.001), 4th (*p* < 0.01) and the 5th (*p* < 0.05) day of trial. However, rapamycin pre-treatment at the dose 3 mg/kg significantly decreased the number of errors in acquisition learning trials on the 1st (*p* < 0.001), 2nd (*p* < 0.01), 3rd (*p* < 0.01), 4th (*p* < 0.01) and the 5th (*p* < 0.05) days. Rapamycin pre-treatment at the dose 10 mg/kg decreased the number of errors in acquisition learning trials only on the 1st (*p* < 0.001), 2nd (*p* < 0.01) and 3rd (*p* < 0.001) days, but on the 4th and 5th days did not (*p* > 0.05) (Figure 2D). The results showed that there were no significant differences between neonatal ethanol and rapamycin groups compared with the controls in the errors during the acquisition of the Barnes maze task (*p* > 0.05).

In the acquisition learning trial, in both the male and female rats, rapamycin alone in both doses (3 mg/kg and 10 mg/kg) did not change the outcome of the undertaken experiments (*p* > 0.05).

### 3.2. The Influence of Rapamycin Pre-Treatment before Every Ethanol Administration during PND4–9 on the Spatial Memory in the Barnes-Maze Task in Adult (PND65) Male and Female Rats

During the probe trial of the Barnes maze test (PND65), a three-way ANOVA indicated significant differences between pre-treatment of rapamycin (F(2,84) = 36.11; *p* < 0.001), ethanol treatment (ethanol effect) (F(1,82) = 71.93; *p* < 0.001) and sex (F(1,84) = 18.27; *p* < 0.001). Three-way ANOVA also showed statistically significant interactions between: ethanol treatment x rapamycin pre-treatment (F(2,84) = 20.77; *p* < 0.001), ethanol treatment x sex (F(1,82) = 7.992; *p* < 0.01), but no interactions between ethanol treatment x rapamycin pre-treatment (F(2,84) = 20.77; *p* > 0.05) and rapamycin pre-treatment x ethanol treatment x sex (F(2,84) = 1.494; *p* < 0.05). With regard to number of errors, three-way ANOVA indicated significant differences between pre-treatment of rapamycin (F(2,84) = 24.50; *p* < 0.001), ethanol treatment groups (ethanol effect) (F(1,82) = 57.26; *p* < 0.001) and sex (F(1,84) = 15.22; *p* < 0.001). Three-way ANOVA also showed statistically significant interactions between: ethanol treatment x rapamycin pre-treatment (F(2,84) = 9.752; *p* < 0.001), ethanol treatment x sex (F(1,82) = 6.688; *p* < 0.05), but no interactions between ethanol treatment x rapamycin pre-treatment (F(2,84) = 2.376; *p* > 0.05) and rapamycin pre-treatment x ethanol treatment x sex (F(2,84) = 0.2477; *p* < 0.05).

Three-way ANOVA indicated that there was a main effect of sex interaction with other factors; therefore, separate analysis of the male and female data was performed.

**Figure 3:**
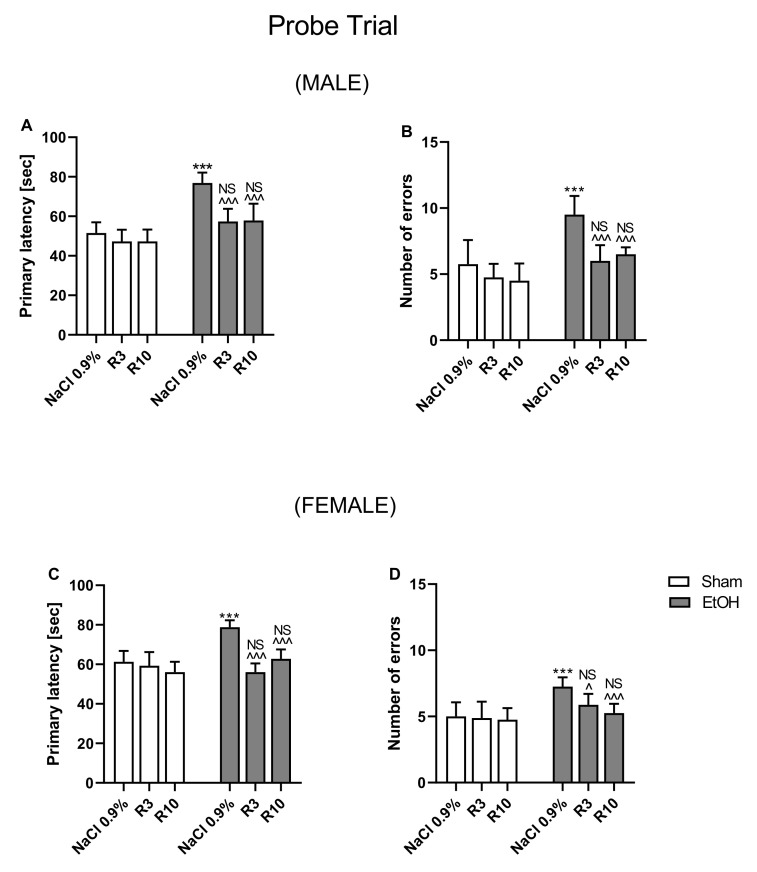
The influence of rapamycin pre-treatment (3 and 10 mg/kg, i.p.) during neonatal period before every ethanol administration (PND 4–9) on the spatial memory impairments (probe trail) in adult (PND66) male and female rats in the Barnes maze task. Primary latency (**A**,**C**) and number of errors (**B**,**D**) are expressed as mean ± SEM of 8 subjects per group. *** *p* < 0.001 vs. SI; ^ *p* < 0.05, ^^^ *p* < 0.001vs. EtOH**,** NS—nonsignificant vs. the non-alcohol treated controls, R3–rapamycin 3 mg/kg; R10–rapamycin 10 mg/kg, SI–shame intubation (i.g.); EtOH–ethanol intubation (i.g.).

Male: During the probe trial of the Barnes maze test (PND65), a two-way ANOVA indicated significant differences between pre-treatment of rapamycin (F(2,42) = 29.76; *p* < 0.001), treatment groups (ethanol effect) (F(1,42) = 106.2; *p* < 0.001) and interactions between these two factors (F(2,42) = 5.746; *p* < 0.001) in primary latency in male rats. Post-hoc analysis (Bonferroni’s multiple comparisons test) showed statistically significant differences between groups in primary latency (Figure 3A). Ethanol administrated for 5 days (PND 4–9) at the dose 5 g/kg (22.66% *v*/*v*, i.g.) increased primary latency (*p* < 0.001) in reaching the escape box. Rapamycin (3 and 10 mg/kg) pre-treatment given before every ethanol administration decreased the primary latency (*p* < 0.001). The results showed that there were no significant differences between neonatal ethanol and rapamycin groups in the primary latency during the probe trial of the Barnes maze task (*p* > 0.05) as compared with the controls.

With regard to number of errors, a two-way ANOVA showed statistically significant differences between pre-treatment groups (F(2,42) *=* 15.57; *p* < 0.001), treatment groups (F(1,42) *=* 39.77; *p* < 0.001) and pre-treatment x treatment interactions (F(2,42) *=* 4.007; *p* < 0.05) in male rats. Post-hoc analysis (Bonferroni’s multiple comparisons test) demonstrated statistically significant differences between the groups of male rats in the number of committed errors (Figure 3B). Ethanol administrated for 5 days (PND 4–9) at the dose 5 g/kg (22.66% *v*/*v*, i.g.) significantly increased the number of errors (*p* < 0.001) committed in reaching the escape box. However, rapamycin pre-treatment before every ethanol administration at the dose of 3 mg/kg (*p* < 0.001) and 10 mg/kg (*p* < 0.001) decreased the number of errors committed by the rats in reaching the escape box. The results showed that there were no significant differences between neonatal ethanol and rapamycin groups in the committed errors during the probe trial of the Barnes maze task (*p* > 0.05 compared to the controls).

Female: During the probe trial conducted on the 6th day of experiment (PND65), a two-way ANOVA indicated significant differences between pre-treatment of rapamycin groups (F(2,42) *=* 26.39; *p* < 0.001), between treatment groups (ethanol effect) (F(1,42) *=* 21.53; *p* < 0.001) and significant interactions between these two factors (F(2,42) *=* 15.84; *p* < 0.001) in primary latency. Post-hoc analysis (Bonferroni’s multiple comparisons test) also demonstrated statistically significant differences between the groups in primary latency (Figure 3C). The analysis revealed that ethanol administrated for 5 days (PND 4–9) at the dose 5 g/kg (22.66% *v*/*v*, i.g.) increased the primary latency (*p* < 0.001) of the rats in reaching the escape box. In contrast, rapamycin pre-treatment decreased the primary latency at the dose of 3 mg/kg and 10 mg/kg (*p* < 0.001). The results showed that there were no significant results between neonatal ethanol and rapamycin groups with result in the controls in the primary latency during the probe trial of the Barnes maze task (*p* > 0.05).

With regard to number of committed errors, a two-way ANOVA showed statistically significant differences between pre-treatment groups (F(2,42) *=* 12.82; *p* < 0.001), treatment groups (F(1,42) *=* 30.42; *p* < 0.001) and pre-treatment x treatment interactions (F(2,42) *=* 9.026, *p* < 0.001). Post-hoc analysis (Bonferroni’s multiple comparisons test) uncovered statistically significant differences between the groups of female rats in term of number of committed errors (Figure 3D). Ethanol administrated for 5 days (PND 4–9) at the dose 5 g/kg (22.66% *v*/*v*, i.g.) increased significantly the number of errors (*p* < 0.001) made in reaching the escape box. However, rapamycin pre-treatment at the dose of 3 mg/kg (*p* < 0.05) and 10 mg/kg (*p* < 0.001) before every ethanol administration decreased the number of errors committed by the female rats in reaching the escape box. The results showed that there were no significant results between neonatal ethanol and rapamycin groups with result in the controls in the committed errors during the probe trial of the Barnes maze task (*p* > 0.05).

In the probe trial, in both the male and female rats, rapamycin alone in both doses (3 mg/kg and 10 mg/kg) did not change the outcome of the undertaken experiments (*p* > 0.05).

### 3.3. The Influence of Rapamycin Pre-Treatment before Every Ethanol Administration during PND4-9 on the Reversal Learning in the Barnes-Maze Task in Adult (PND 66–69) Male and Female Rats

Reversal learning of spatial memory was evaluated by a decrease in the latency time and the number of errors to reach the escape box for five consecutive days (PND61–65). A four-way ANOVA with repeated measurements showed a significant effect of rapamycin pre-treatment (F(2,464) *=* 33.18; *p* < 0.001), ethanol treatment (F(1,464) *=* 9.933; *p* < 0.01), testing day (F(4,464) *=* 114.5.; *p* < 0.001) and significant effect of sex (F(1,464) *=* 4.89; *p* < 0.05). Analysis also showed significant interactions between sex x testing day (F(4,464) *=* 12.41; *p* < 0.001), sex x ethanol (F(1,464) *=* 27.53; *p* < 0.001) and sex x rapamycin pre-treatment (F(2464) *=* 3.147); *p* < 0.05) in female adult rats (PND61–65).

In the number of primary errors committed by rats, a four-way ANOVA with repeated measurements showed a significant effect of rapamycin pre-treatment (F (2,464) *=* 33.18; *p* < 0.001), ethanol treatment (F(1,464) *=* 9.933; *p* < 0.01), testing day (F(4,464) *=* 114.5; *p* < 0.001) and a significant effect of sex (F(1,464) *=* 4.89). Analysis also showed significant interaction between sex x testing day (F(4,464) *=* 12.41; *p* < 0.001), sex x ethanol (F(1,464) *=* 27.53; *p* < 0.001) and significant interactions between sex x rapamycin pre-treatment (F(2,464) *=* 3.147); *p* < 0.05) in adult rats (PND61–65).

Four-way ANOVA indicated that there was a significant effect of sex interaction with other factors; therefore, separate analysis of the male and female data was performed.

**Figure 4:**
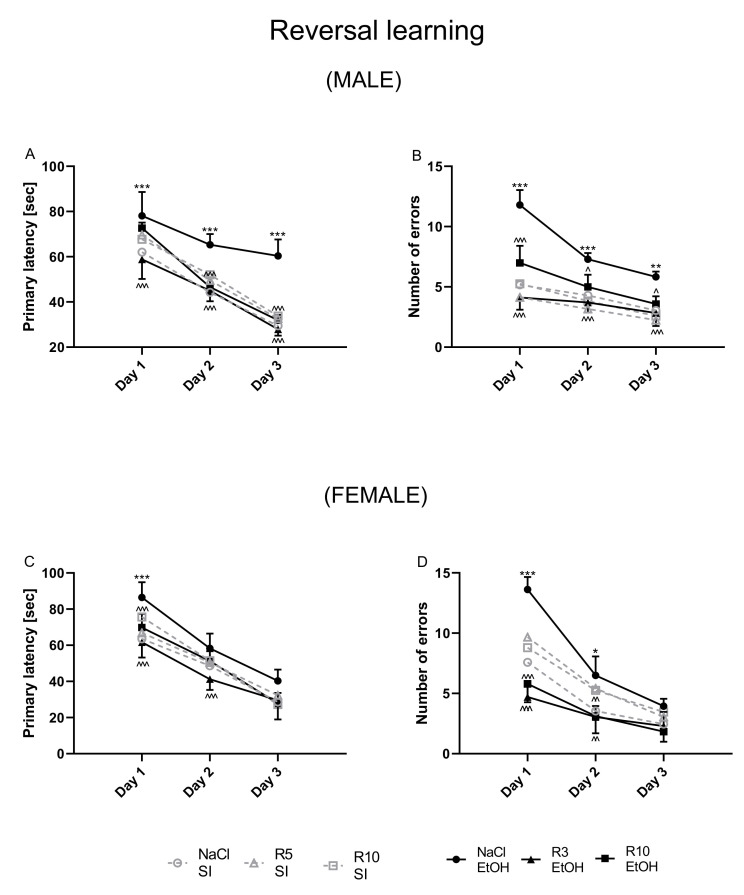
The influence of rapamycin pre-treatment (3 and 10 mg/kg, i.p.) during neonatal period before every ethanol administration (PND4–9) on the reversal learning impairments in adult (PND67–69) male and female rats in the Barnes maze task. Primary latency (**A**,**C**) and number of errors (**B**,**D**) are expressed as mean ± SEM of 8 subjects per group (R 3.6.0. for Windows). * *p* < 0.05; ** *p* < 0.01; *** *p* < 0.001 vs. SI; ^ *p* < 0.05; ^^ *p* < 0.01; ^^^ *p* < 0.001 vs. EtOH. R3–rapamycin 3 mg/kg; R10–rapamycin 10 mg/kg, SI–shame intubation (i.g.); EtOH–ethanol intubation (i.g.).

Male: Reversal learning trials were performed one day after probe trial. A two-way ANOVA with repeated measures showed a significant effect of day of reversal learning (F(2,126) *=* 270; *p* < 0.001) and group of rats’ effect (F(5,126) *=* 38.38; *p* < 0.001) and interaction between these factors (F(10,126) *=* 3.157; *p* < 0.01) in male adult rats (PND67–69). Moreover, the post-hoc test demonstrated that latency to reach the escape box was impaired following neonatal ethanol exposure on the 1st day (*p* < 0.001), 2nd (*p* < 0.001) and 3rd (*p* < 0.001) days of the trial. Pre-treatment with rapamycin (3 mg/kg, i.p.), decreased the primary latency on the 1st (*p* < 0.001), 2nd (*p* < 0.001) and 3rd (*p* < 0.001) days of reversal learning trials. In male rats, pre-treatment with rapamycin (10 mg/kg) failed to decrease the effect of neonatal alcohol on the 1st day of reversal learning (*p* > 0.05), but significantly decreased the primary latency to reach the escape box on the 2nd and 3rd day (*p* < 0.001) of reversal learning (Figure 4A). The results showed that there were no significant differences between neonatal ethanol and rapamycin groups in the primary latency during the reversal learning of the Barnes maze task (*p* > 0.05), as compared to the controls.

In the number of primary errors committed by rats, a two-way ANOVA with repeated measures indicated significant effect of day of reversal learning (F(2,126) *=* 69.56; *p* < 0.001), group of rats (F(5,126) *=* 58.75; *p* < 0.001) and interaction between these two factors in reversal learning (F(10,126) *=* 4.596 *p* < 0.001) in male adult rats (PND67–69). The post-hoc test showed that the ethanol exposed group committed significantly more errors in finding the escape box than did the control group on the 1st (*p* < 0.001), 2nd (*p* < 0.001) and 3rd (*p* < 0.01) day trials. Rapamycin pre-treatment at the dose of 3 mg/kg more significantly reversed this ethanol effect (on the 1st, 2nd and 3rd day; *p* < 0.001) than at the dose of 10 mg/kg (on the 1st day (*p* < 0.001), 2nd day (*p* < 0.05) and 3rd day (*p* < 0.05)) (Figure 4B) in adult male rats (PND67–69). The results showed that there were no significant differences between neonatal ethanol and rapamycin groups in the committed errors during the reversal learning of the Barnes maze task (*p* > 0.05) compared to the controls.

Female: Reversal learning trials were performed one day after probe trial. A two-way ANOVA with repeated measures uncovered the significant effect of days of reversal learning (F(2,126) *=* 372.5; *p* < 0.001), group of rats (F(5,126) *=* 16.98; *p* < 0.001) and interaction between groups x days (F(10,126) *=* 2.403 *p* < 0.05). In addition, the post-hoc test (Bonferroni’s multiple comparisons) showed that latency to reach the escape box was impaired following neonatal ethanol exposure (5 days (PND4–9) at the dose 5 g/kg, 22.66% *v*/*v*, i.g. on the 1st day (*p* < 0.001), but not on the 2nd (*p* > 0.05) and 3rd (*p* > 0.05) days of the trial. Pre-treatment with rapamycin (3 mg/kg, i.p.) equally reduced the primary latency on the 1st and 2nd (*p* < 0.001) but did not do so on the 3rd (*p* > 0.05) day of reversal learning trials. In addition, pre-treatment with rapamycin (10 mg/kg) reduced the primary latency only on the 1st (*p* < 0.001), but not the 2nd and 3rd (*p* > 0.05) days of reversal learning in female adult rats (PND65–67) (Figure 4C). The results showed that there were no significant differences between neonatal ethanol and rapamycin groups in the primary latency during the reversal learning of the Barnes maze task (*p* > 0.05) compared with the controls.

With regard to the number of errors committed by the female rats, a two-way ANOVA with repeated measures indicated the significant effect of days (F(2,126 *=* 164.1; *p* < 0.001), groups (F(5,126) *=* 31.78; *p* < 0.001) and groups x days interactions (F (10,126) *=* 5.745 *p* < 0.001) in the reversal learning task. The post-hoc test (Bonferroni’s multiple comparisons) demonstrated that the ethanol exposed group committed much more errors in finding the escape box than did the control group on the 1st (*p* < 0.001) and 2nd (*p* < 0.05), but not on the 3rd (*p* > 0.05) day of trial. However, rapamycin in both doses of 3 mg/kg and 10 mg/kg as pre-treatment before every ethanol administration significantly decreased the number of errors in reversal learning trials on the 1st (*p* < 0.001) and 2nd (*p* < 0.01), but not on the on-3rd day (*p* > 0.05) days of the reversal learning (Figure 4D) in adult female rats (PND67–69). The results showed that there were no significant differences between neonatal ethanol and rapamycin groups in the committed errors during the reversal learning of the Barnes maze task (*p* > 0.05) as compared with the controls.

In the reversal learning trial, in both the male and female rats, rapamycin alone in both doses (3 mg/kg and 10 mg/kg) did not change the outcome of the undertaken experiments.

### 3.4. The Effects of Rapamycin Pre-Treatment before Every Ethanol Administration during PND4–9 on the Rewarding Effects of Ethanol in Adult Male Rats (70PND) in CPP Test

**Figure 5:**
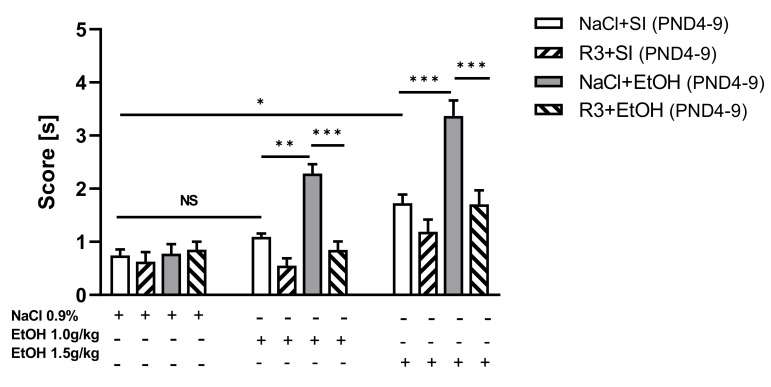
The influence of rapamycin pre-treatment (3 mg/kg, i.p.) during neonatal period before every ethanol administration on the development of CPP induced by ethanol (1.0 g/kg; 1.5 g/kg) in adult male rats (PND60–70). Data are shown as post-conditioning minus pre-conditioning time (sec) spent in the drug-associated compartment. Results are expressed as mean ± SEM of 8 subjects per group (GraphPad version 8.0 for Windows). * *p* < 0.05 ** *p* < 0.01; *** *p* < 0.001. R3–rapamycin 3 mg/kg; SI–shame intubation (i.g.); EtOH–ethanol intubation (i.g.).

Three-way ANOVA analysis indicated statistically significant differences between rapamycin pre-treatment (F(1,84) *=* 43.23; *p* < 0.001), ethanol treatment (ethanol effect) (F(1,84) *=* 36.93; *p* < 0.001) and conditioning by ethanol during CPP (F(2,84) *=* 46.41; *p* < 0.001). Three-way ANOVA also showed statistically significant interactions between: ethanol dose x rapamycin pre-treatment (F(2,84) *=* 10.22; *p* < 0.001), ethanol dose x ethanol treatment (F(2,82) *=* 6.709; *p* < 0.01), ethanol treatment x rapamycin pre-treatment (F(1,84) *=* 8.050; *p* < 0.01) and ethanol dose x ethanol treatment x rapamycin pre-treatment (F(2,84) *=* 3.582; *p* < 0.05). The post-hoc analysis revealed that ethanol at the dose of 1.0 g/kg induced a significant place preference only in adult male rats (PND70) that received ethanol during the neonatal period (*p* < 0.01). However, ethanol at the dose of 1.5 g/kg given during acquisition of CPP induced a significant CPP in both animals that previously received ethanol (*p* < 0.001) and in the control group (*p* < 0.05). Moreover, the effect of ethanol at the dose 1.5g/kg was significantly higher than at the dose 1.0 g/kg (*p* < 0.01). This result suggests that a dose dependent effect exists with regard to the rewarding effect of ethanol in animals that received ethanol during neonatal period (Figure 5). Interestingly, post-hoc test indicated that rapamycin pre-treatment (3 mg/kg) during neonatal period (PND4–9) significantly decreased the ethanol-induced CPP after 8 days of conditioning in both doses: 1.0 g/kg and 1.5 g/kg (*p* < 0.001). These results were expressed as significantly less time spent in the ethanol-associated compartment during the post-conditioning test (Figure 5). As seen in Table 2, ethanol (1.0 and 1.5 g/kg) given during the development of CPP had no effect on the locomotor activity of rats on the test day that received ethanol during neonatal period (F(2,21) *=* 2.405; *p =* 0.114).

### 3.5. The Effects of Rapamycin Pre-Treatment on the Expression of the Synaptosomal GluN2B Levels in the Hippocampus and Prefrontal Cortex in Adult Male and Female Rats Exposed to the Ethanol during Neonatal Period

Three-way ANOVA in the prefrontal cortex indicated significant differences between pre-treatment of rapamycin (F(2,84) *=* 18.22; *p* < 0.001), ethanol treatment (ethanol effect) (F(1,82) *=* 16.37; *p* < 0.001) and sex (F(1,84) *=* 8.138; *p* < 0.001). Three-way ANOVA also showed statistically significant interactions between: ethanol treatment x rapamycin pre-treatment (F(2,84) *=* 37.43; *p* < 0.001), but no interactions between rapamycin pre-treatment x sex (F(2,84) *=* 0.8617; *p* > 0.05); ethanol treatment x sex (F(1,82) *=* 0.7859; *p* > 0.05) and rapamycin pre-treatment x ethanol treatment x sex (F(2,84) *=* 0.8290; *p* > 0.05) in synaptosomal GluN2B expression.

Three-way ANOVA in the hippocampus indicated significant differences between pre-treatment of rapamycin (F(2,84) *=* 10.47; *p* < 0.001), ethanol treatment (ethanol effect) (F(1,82) *=* 9.789; *p* < 0.01) and sex (F(1,84) *=* 5.723; *p* < 0.05). Three-way ANOVA also showed statistically significant interactions between: ethanol treatment x rapamycin pre-treatment (F(2,84) *=* 22.70; *p* < 0.001) and ethanol treatment x sex (F(1,82) *=* 21.37; *p* < 0.001) but no interactions between rapamycin pre-treatment x sex (F(2,84) *=* 0.1.346; *p* > 0.05) and rapamycin pre-treatment x ethanol treatment x sex (F(2,84) *=* 0.1521; *p* > 0.05) in synaptosomal GluN2B expression.

Three-way ANOVA in the striatum indicated significant differences between pre-treatment of rapamycin (F(2,84) *=* 13.75; *p* < 0.001), ethanol treatment (ethanol effect) (F(1,82) *=* 27.68; *p* < 0.001) and sex (F(1,84) *=* 6.856; *p* < 0.05). Three-way ANOVA also showed statistically significant interactions between: ethanol treatment x rapamycin pre-treatment (F(2,84) *=* 6.637; *p* < 0.01), but no interactions between rapamycin pre-treatment x sex (F(2,84) *=* 0.9431; *p* > 0.05); ethanol treatment x sex (F(1,82) *=* 0.01236; *p* > 0.05) and rapamycin pre-treatment x ethanol treatment x sex (F(2,84) *=* 0.1270; *p* > 0.05) in synaptosomal GluN2B expression.

Three-way ANOVA indicated that there is an effect of sex interaction with other factors; therefore, separate analysis of the male and female data was performed.

Two-way ANOVA in male rats indicated significant differences between rapamycin pre-treatment (F(2,42) *=* 5.211; *p* < 0.01), ethanol treatment (F(1,42) *=* 24.70; *p* < 0.001) and interactions between these two factors (F(2,42) *=* 9.574; *p* < 0.001) in the hippocampus. Two-way analysis also indicated significant differences between rapamycin pre-treatment (F(2,42) *=* 9.50; *p* < 0.001) in the prefrontal cortex and in the striatum (F(2,42) *=* 3.277, *p* < 0.05) and between ethanol treatment in the prefrontal cortex (F(1,42) *=* 9.198; *p* < 0.01) and in the striatum (F(1,42) *=* 21.57); *p* < 0.01) and interactions between these two factors in the prefrontal cortex (F(2,42) *=* 18.14; *p* < 0.001) and striatum (F(2,42) *=* 15.39; *p* < 0.01) in synaptosomal GluN2B expression. Post-hoc tests (Bonferroni) demonstrated that neonatal ethanol administration decreased the synaptosomal GluN2B levels in the hippocampus (*p* < 0.001) and prefrontal cortex (*p* < 0.001). Rapamycin pre-treatment at the dose of 3 mg/kg reversed the alcohol-induced reduction in the synaptosomal GluN2B levels both in the hippocampus (*p* < 0.001) and prefrontal cortex (*p* < 0.001). Rapamycin at the dose of 10 mg/kg, given before ethanol administration also reversed the alcohol-induced reduction in the synaptosomal GluN2B levels in the hippocampus (*p* < 0.01) and prefrontal cortex (*p* < 0.001).

**Figure 6:**
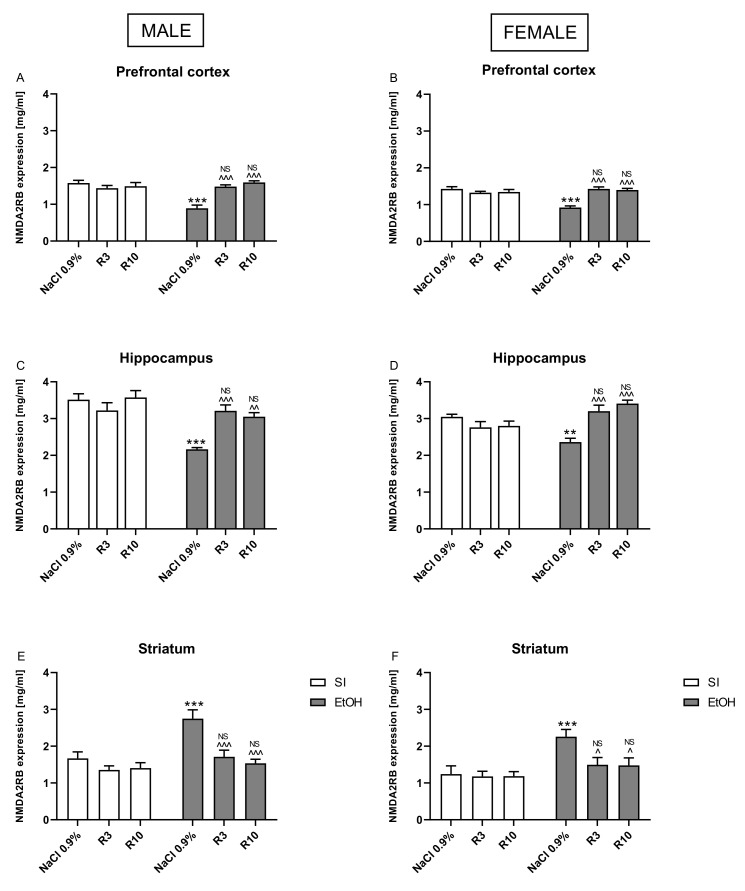
The influence of rapamycin pre-treatment (3 and 10 mg/kg, i.p.) during neonatal period before every ethanol administration (PND4–9) on the expression of GluN2B subunit of NMDA receptor in adult (PND69) male and female rats in the prefrontal cortex (**A**,**C**) and hippocampus (**B**,**D**). Striatum: (**E**,**F**). Results are expressed as mean ± SEM of 8 subjects per group. ** *p* < 0.01; *** *p* < 0.001 vs. SI; ^ *p* < 0.05; ^^ *p* < 0.01; ^^^ *p* < 0.001 vs. EtOH. NS–nonsignificant vs. the non-alcohol treated controls; EtOH R3–rapamycin 3 mg/kg; R10–rapamycin 10 mg/kg, SI–shame intubation (i.g.); EtOH–ethanol intubation (i.g.).

Post-hoc analysis also showed that that neonatal ethanol administration significantly increased the synaptosomal GluN2B level in the striatum (*p* < 0.001). Rapamycin pre-treatment at the dose of 3 mg/kg (*p* < 0.001) and at the dose 10 mg/kg (*p* < 0.001) before ethanol administration in both doses normalized the GluN2B expression to the values of the corresponding control groups (Figure 6A,C,E). The results showed that there were no significant differences between neonatal ethanol and rapamycin groups in synaptosomal GluN2B levels (*p* > 0.05) as compared the controls.

Two-way ANOVA in female rats indicated significant differences between rapamycin pre-treatment (F(2,42) *=* 3.362; *p* < 0.05), ethanol treatment (F(1,42) *=* 4.297; *p* < 0.05) and interactions between these two factors (F(2,42) *=* 19.46; *p* < 0.001) in the hippocampus. In the prefrontal cortex, two-way analysis also indicated significant differences between rapamycin pre-treatment (F(2,42) *=* 9.619; *p* < 0.001), ethanol treatment (ethanol effect) (F(1,42) *=* 7.370; *p* < 0.01) and interactions between these two factors (F(2,42) *=* 21.06; *p* < 0.001) in the synaptosomal GluN2B levels. Furthermore, in the striatum, two-way analysis indicated significant differences between rapamycin pre-treatment (F(2,42) *=* 3.398; *p* < 0.05), ethanol treatment (ethanol effect) (F(1,42) *=* 27.81; *p* < 0.05) and interactions between these two factors (F(2,42) *=* 3.297; *p* < 0.001) in the synaptosomal GluN2B levels.

Post-hoc tests in female rats showed that ethanol administration during the neonatal period decreased the synaptosomal GluN2B levels in the hippocampus (*p* < 0.01) and prefrontal cortex (*p* < 0.001). Rapamycin pre-treatment at the dose of 3 mg/kg reversed the alcohol-induced reduction in the synaptosomal GluN2B levels both in the hippocampus (*p* < 0.001) and prefrontal cortex (*p* < 0.001). In addition, rapamycin at the dose of 10 mg/kg given before ethanol administration reversed ethanol-induced reduction in the synaptosomal GluN2B levels in the hippocampus and prefrontal cortex (*p* < 0.001). Post-hoc analysis show that that neonatal ethanol administration significantly increased the synaptosomal GluN2B level in the striatum (*p* < 0.01), while rapamycin pre-treatment before ethanol administration in both doses (3 mg/kg (*p* < 0.05) and 10 mg/kg (*p* < 0.05)) normalized the GluN2B expression to the values of the corresponding control group (Figure 6B,D,F). The results showed that there were no significant results between neonatal ethanol and rapamycin groups with result in the controls in synaptosomal GluN2B levels (*p* > 0.05).

In the GluN2B levels in both the male and female rats, rapamycin alone in both doses (3 mg/kg and 10 mg/kg) did not change the outcome of the undertaken experiments.

## 4. Discussion

The present study demonstrated that rats exposed to ethanol during the neonatal period show behavioral deficits in a spatial learning and memory and in a spatial reversal learning in the Barnes maze task. There was a significant impact of sex on behavior in the probe trial and reversal learning but this factor was less pronounced in the acquisition learning. Furthermore, adult male rats exposed to ethanol during PND4–9 were more sensitive to rewarding effects of ethanol as measured in the CPP test. Western blot analysis showed a decrease of the GluN2B subunit of NMDA receptor in the prefrontal cortex and hippocampus and an increase of this subunit in the striatum in adult rats that were ethanol-treated in the neonatal period. Rapamycin pretreatment before every ethanol administration during this time mitigated learning and memory impairment, prevented alcohol addiction and normalized the GluN2B expression to the values of the corresponding control group. Thus, our findings indicate that rapamycin, an mTORC1 inhibitor, can prevent the deleterious effects of ethanol on the developing brain when given before ethanol exposure to neonatal rats.

### 4.1. Rapamycin Prevents Ethanol-Induced Spatial Memory Impairment and Reversal Learning

The current results agree with clinical [44,45] and animal findings [46,47] that showed deficits in spatial memory in adults associated with neonatal ethanol exposure. However, few studies have emphasized sex differences in memory performance in adult animals exposed to ethanol during perinatal period [48,49,50,51]. One study, for example, demonstrated that even a single exposure to ethanol in the early postnatal period induced memory impairments in adult male, but not female animals [52]. Other studies reported that three days of ethanol exposure (PND7–9) induced deficits only in adult male animals [48,49], but longer ethanol exposure (over 5–6 days) promoted memory impairments in both male and female rats [47,50,53]. We saw deleterious effect of ethanol on memory in both sexes.

Our results demonstrated that ethanol treated rats made more errors and spent more time in finding the hidden escape box in the Barnes maze task during the acquisition learning, in the probe trial and reversal learning. Furthermore, we showed that ethanol given during the neonatal period was able to impair reversal learning in adult rats Reversal learning is representative of flexibility and adaptability to a changing environment [54]. Such impairments in cognition are a component of alcohol addiction and are related to the difficulty that alcoholics have in changing their behavior, even when alcohol use is associated with negative consequences [55]. In our study, the spatial processing impairment (observed in the probe) could suggest deleterious effects in the ability of these PND4–9 ethanol-treated rats to use spatial cues in reversal-learning, even though their cognitive flexibilities could be intact. Thus, spatial processes impairment is probably responsible for all the deficits observed in these animals. Considering the aforementioned, it can be concluded that rats that received ethanol over PND4–9 were not able to remember the spatial cues that can allow them to find the escape box. Thus, it can be said that the reversal learning deficits in adult rats following repeated ethanol administration during neonatal period may have been due to spatial learning rather than cognitive flexibility impairments. We should note that animals with reversal learning deficits usually show perpetuation of drug-seeking behavior and relapse [56,57,58,59]. In our experiment, memory impairment effects were not observed in animals that received rapamycin, the mTORC1 inhibitor, before ethanol administration during the neonatal period.

### 4.2. Rapamycin Prevents Rewarding Effect of Ethanol

Our data also show that early life ethanol exposure facilitated the induction of CPP by ethanol. Such outcome is based on the observation that ethanol at the dose of 1.0 g/kg induced CPP in these animals, whereas no rewarding effect was found (at this dose) in the control group. Furthermore, ethanol at the higher dose of 1.5 g/kg induced more rewarding effect in the ethanol-exposed rats (*p* < 0.001) than in the control rats (*p* < 0.05). These data suggest that neonatal animals exposed to alcohol have enhanced sensitivity to the rewarding effect of ethanol in adulthood. Likewise, increased sensitivity in the CPP, induced by a psychostimulant drug (amphetamine or cocaine) was observed in prenatal alcohol exposure rats upon reaching adulthood [60]. It should be noted that such effects were observed at the ethanol doses that did not induce significant changes in the animal’s locomotor activity. Thus, our results are in agreement with previous work demonstrating that prenatal ethanol exposure increases alcohol preference in later life in humans [11] and in rodents [61,62] and confirm that the drug’s rewarding properties not associated with the sensitivity to its locomotor stimulation effect [12,63,64]. In our study, rapamycin, the mTORC1 inhibitor, given before ethanol administration during the neonatal period prevented the sensitivity to rewarding effects of ethanol in adulthood. Our study is supported, at least partially, by the findings of Neasta et al. [65] that revealed that alcohol administration activated the mTOR signaling cascade in the nucleus accumbens of adult mice. They also demonstrated that rapamycin, an inhibitor of mTORC1, decreased the expression of alcohol-induced place preference, as well as excessive alcohol intake.

The changes in learning processes induced by alcohol administration in the neonatal period can also have had significant impact in the results obtained in the CPP test. The drug effect on reward pathways creates learning signals to facilitate learning about stimuli in the environment associated with the drug [66,67]. Such memory activations (alcohol impairs encoding and enhances early consolidation of memory for beverage-related stimuli) after repeated environmental exposure may induce craving in the absence of drugs that is followed by alcohol-seeking and consumption [68]. Thus, our studies suggest that neonatal exposure to ethanol increases propensity to develop alcoholism and rapamycin prevented this.

### 4.3. Rapamycin Prevents Ethanol Induced Changes in GluN2B Subunit Expression

The results of our study indicate that neonatal ethanol exposure downregulated the expression of the GluN2B subunit of the NMDA receptor in the prefrontal cortex and hippocampus in adult male and female rats. Our data confirms and extends previous findings suggesting that even a single binge-like ethanol exposure during the brain growth spurt (equivalent to the third trimester in human) is sufficient to significantly reduce the GluN2B expression, at least, in the adult male hippocampus [52].

Ethanol is a well-known antagonist of NMDA receptors and prenatal ethanol exposure alters the NMDA receptor subunits expression in the adult brain. The GluN2B subunit is particularly expressed during early brain development and is sensitive to alcohol exposure. Earlier studies suggested that ethanol administration, both parental and postnatal, can induce reduction in NMDA receptor density or decrease the amount of GluN2B subunits [19,69,70]. In the hippocampus, the GluN2B subunit is highly expressed and is known to be important in memory formation [71]. Here, the GluN2B is associated with synaptic plasticity and hippocampal long-term potentiation (LTP) [72]. The decrease in synaptic GluN2B levels and function may lead to the LTP deficits seen in alcohol prenatal exposed mice [73,74]. Thus, our study confirms the memory impairment associated with downregulation of the GluN2B subunit in the Barnes maze task in adult rats exposed to ethanol during the neonatal period.

Furthermore, our study reveals an increase in GluN2B expression in the striatum in adult rats exposed neonatally to alcohol. The dorsal striatum is a subcortical brain region important for proper motor function [75], but also plays a key role in instrumental learning, habit formation [76] and drug addiction [77]. Activation of NMDA receptors is required for the induction of LTP in various brain regions such as the hippocampus and cortex, as well as in the dorsal striatum [78,79]. Thus, our results are consistent with previously published that show that repeated ethanol administrations and withdrawal facilitate LTP induction via upregulation of NR2B subunit of the NMDA receptor activity [80]. Such aberrant plasticity may contribute to long-lasting neuroadaptations that are associated with such pathological ethanol-related behaviors as the development and maintenance of excessive ethanol drinking in adulthood.

### 4.4. Potential Mechanism of Rapamycin

The present study confirms the involvement of the mTOR pathway in disorders associated with neonatal ethanol exposure [81]. Although we did not evaluate the influence of ethanol on the mTOR signaling pathway, pretreatment with rapamycin before ethanol administration in neonatal rats mitigated the negative effects of neonatal ethanol exposure on rat behavior and neurochemical evaluation. These data support the previous finding that gestational binge alcohol exposure dysregulates the fetal hippocampal mTOR system, has an impact on mTORC1 signaling, which plays essential roles in brain development and alters the proteins that complex with hippocampal mTOR, the functions of which have also been implicated in brain developmental processes [33,82]. Furthermore, it has been previously indicated that inhibition of GluN2B in the orbitofrontal cortex attenuates alcohol-dependent mTORC1 activation, alcohol seeking and habitual responding for alcohol. These data imply that the GluN2B/mTORC1 axis in the orbitofrontal cortex drives alcohol seeking and habit [83]. The exact mechanism of such interactions needs further evaluation. However, our work reveals that mTORC1–GluN2B interactions play an important role in learning and memory and drive seeking behavior in adult rats that received ethanol during neonatal period.

## 5. Conclusions

We conclude that the mTOR signaling pathway has responsibility for the effects of alcohol on brain development during the neonatal period and subsequent changes in adult brain. As the mTOR signaling pathway is involved in axonal regeneration, dendritic arborization, synaptic plasticity, cellular growth and autophagy, the mTOR signaling pathway and fetal alcohol disorders are probably related. More work, however, is needed to untangle the complexity of the mTOR signaling pathway to understand the effect of alcohol during fetal growth on learning and memory impairment and sensitivity to alcohol abuse, as well as alcohol relapse in adulthood.

## Figures and Tables

**Table 1:** Experimental groups. SI–shame intubation (i.g.); EtOH–ethanol intubation (i.g.); R3–rapamycin 3 mg/kg (i.p.); R10–rapamycin 10 mg/kg (i.p.).

**Experiment 1**	**Sex**	**N**
1. SI + 0.9%NaCl	male, female	8/8
2. SI + R3	male, female	8/8
3. SI + R10 4. EtOH + 0.9%NaCl 5. EtOH + R3 6. EtOH +R10	male, female male, female male, female male, female	8/8 8/8 8/8 8/8
**Experiment 2**	**Sex**	**N**
1. SI + 0.9%NaCl	male	8
2. SI + R3	male	8
3. EtOH + 0.9%NaCl 4. EtOH + R3	male male	8 8
**Experiment 3**	**Sex**	**N**
1. SI + 0.9%NaCl	male, female	8/8
2. SI + R3	male, female	8/8
3. SI + R10 4. EtOH + 0.9%NaCl 5. EtOH + R3 6. EtOH +R10	male, female male, female male, female male, female	8/8 8/8 8/8 8/8

**Table 2:** Effect of early ethanol exposure on ethanol-induced locomotor activity in the CPP test.

Compounds	N	Distance Traveled (m) ± SEM
NaCl 0.9%	8	24.06 ± 1.513 (NS)
EtOH 1.0 g/kg	8	20.94 ± 1.502 (NS)
EtOH 1.5 g/kg	8	21.89 ± 1.688 (NS)

## Data Availability

The data presented in this study are available on request from the corresponding author.

## References

[1] Dörrie N., Föcker M., Freunscht I., Hebebrand J. (2014). Fetal alcohol spectrum disorders. Eur. Child Adolesc. Psychiatry.

[2] Senturias Y., Asamoah A. (2014). Fetal alcohol spectrum disorders: Guidance for recognition, diagnosis, differential diagnosis and referral. Curr. Probl. Pediatr. Adolesc. Health Care.

[3] Jones K.L., Smith D.W. (1973). Recognition of the fetal alcohol syndrome in early infancy. Lancet.

[4] Willoughby K.A., Sheard E.D., Nash K., Rovet J. (2008). Effects of prenatal alcohol exposure on hippocampal volume, verbal learning, and verbal and spatial recall in late childhood. J. Int. Neuropsychol. Soc..

[5] Manji S., Pei J., Loomes C., Rasmussen C. (2009). A review of the verbal and visual memory impairments in children with foetal alcohol spectrum disorders. Dev. Neurorehabilit..

[6] Beninger R.J., Gerdjikov T. (2004). The role of signaling molecules in reward-related incentive learning. Neurotox. Res..

[7] Zhai H.F., Zhang Z.Y., Zhao M., Qiu Y., Ghitza U.E., Lu L. (2007). Conditioned drug reward enhances subsequent spatial learning and memory in rats. Psychopharmacology.

[8] Gould T.J. (2010). Addiction and cognition. Addict. Sci. Clin. Pract..

[9] Goodman J., Packard M.G. (2016). Memory Systems and the Addicted Brain. Front. Psychiatry.

[10] Brancato A., Castelli V., Cavallaro A., Lavanco G., Plescia F., Cannizzaro C. (2018). Pre-conceptional and Peri-Gestational Maternal Binge Alcohol Drinking Produces Inheritance of Mood Disturbances and Alcohol Vulnerability in the Adolescent Offspring. Front. Psychiatry.

[11] Spear N.E., Molina J.C. (2005). Fetal or infantile exposure to ethanol promotes ethanol ingestion in adolescence and adulthood: A theoretical review. Alcohol. Clin. Exp. Res..

[12] Barbier E., Pierrefiche O., Vaudry D., Vaudry H., Daoust M., Naassila M. (2008). Long-term alterations in vulnerability to addiction to drugs of abuse and in brain gene expression after early life ethanol exposure. Neuropharmacology.

[13] Cantacorps L., González-Pardo H., Arias J.L., Valverde O., Conejo N.M. (2018). Altered brain functional connectivity and behaviour in a mouse model of maternal alcohol binge-drinking. Prog. Neuropsychopharmacol. Biol. Psychiatry.

[14] Parker M.O., Evans A.M., Brock A.J., Combe F.J., The M.T., Brennan C.H. (2016). Moderate alcohol exposure during early brain development increases stimulus-response habits in adulthood. Addict. Biol..

[15] Marmiroli P., Cavaletti G. (2012). The glutamatergic neurotransmission in the central nervous system. Curr. Med. Chem..

[16] Mayford M., Siegelbaum S.A., Kandel E.R. (2012). Synapses and memory storage. Cold Spring Harb. Perspect. Biol..

[17] Lüscher C. (2013). Drug-evoked synaptic plasticity causing addictive behavior. J. Neurosci..

[18] Pierrefiche O. (2017). Long Term Depression in Rat Hippocampus and the Effect of Ethanol during Fetal Life. Brain Sci..

[19] Zhang T.A., Hendricson A.W., Wilkemeyer M.F., Lippmann M.J., Charness M.E., Morrisett R.A. (2005). Synergistic effects of the peptide fragment D-NAPVSIPQ on ethanol inhibition of synaptic plasticity and NMDA receptors in rat hippocampus. Neuroscience.

[20] Subbanna S., Basavarajappa B.S. (2020). Postnatal Ethanol-Induced Neurodegeneration Involves CB1R-Mediated β-Catenin Degradation in Neonatal Mice. Brain Sci..

[21] Lloyd B.A., Hake H.S., Ishiwata T., Farmer C.E., Loetz E.C., Fleshner M., Bland S.T., Greenwood B.N. (2017). Exercise increases mTOR signaling in brain regions involved in cognition and emotional behavior. Behav. Brain Res..

[22] Graber T.E., McCamphill P.K., Sossin W.S. (2013). A recollection of mTOR signaling in learning and memory. Learn. Mem..

[23] Saxton R.A., Sabatini D.M. (2017). mTOR Signaling in Growth, Metabolism, and Disease. Cell.

[24] Neasta J., Barak S., Hamida S.B., Ron D. (2014). mTOR complex 1: A key player in neuroadaptations induced by drugs of abuse. J. Neurochem..

[25] Hoeffer C.A., Klann E. (2010). mTOR signaling: At the crossroads of plasticity, memory and disease. Trends Neurosci..

[26] Guertin D.A., Sabatini D.M. (2009). The pharmacology of mTOR inhibition. Sci. Signal..

[27] Luo J. (2014). Autophagy and ethanol neurotoxicity. Autophagy.

[28] Chen G., Ke Z., Xu M., Liao M., Wang X., Qi Y., Zhang T., Frank J.A., Bower K.A., Shi X. (2012). Autophagy is a protective response to ethanol neurotoxicity. Autophagy.

[29] Brady M.L., Diaz M.R., Iuso A., Everett J.C., Valenzuela C.F., Caldwell K.K. (2013). Moderate prenatal alcohol exposure reduces plasticity and alters NMDA receptor subunit composition in the dentate gyrus. J. Neurosci..

[30] Samudio-Ruiz S.L., Allan A.M., Sheema S., Caldwell K.K. (2010). Hippocampal N-methyl-D-aspartate receptor subunit expression profiles in a mouse model of prenatal alcohol exposure. Alcohol. Clin. Exp. Res..

[31] Bayer S.A., Altman J., Russo R.J., Zhang X. (1993). Timetables of neurogenesis in the human brain based on experimentally determined patterns in the rat. Neurotoxicology.

[32] Goodlett C.R., Johnson T.B. (1999). Temporal windows of vulnerability to alcohol during the third trimester equivalent: Why “knowing when” matters. Alcohol and Alcoholism: Effects on Brain and Development.

[33] Lopatynska-Mazurek M., Pankowska A., Gibula-Tarlowska E., Pietura R., Kotlinska J.H. (2021). Rapamycin Improves Recognition Memory and Normalizes Amino-Acids and Amines Levels in the Hippocampal Dentate Gyrus in Adult Rats Exposed to Ethanol during the Neonatal Period. Biomolecules.

[34] Gibula-Tarlowska E., Kotlinska J.H. (2020). Kissorphin improves spatial memory and cognitive flexibility impairment induced by ethanol treatment in the Barnes maze task in rats. Behav. Pharmcol..

[35] Gibula-Tarlowska E., Wydra K., Kotlinska J.H. (2020). Deleterious Effects of Ethanol, Δ(9)-Tetrahydrocannabinol (THC), and Their Combination on the Spatial Memory and Cognitive Flexibility in Adolescent and Adult Male Rats in the Barnes Maze Task. Pharmaceutics.

[36] Marszalek-Grabska M., Gibula-Bruzda E., Bodzon-Kulakowska A., Suder P., Gawel K., Talarek S., Listos J., Kedzierska E., Danysz W., Kotlinska J.H. (2018). ADX-47273, a mGlu5 receptor positive allosteric modulator, attenuates deficits, in cognitive flexibility induced by withdrawal from ’binge-like’ ethanol exposure in rats. Behav. Brain Res..

[37] Gawel K., Labuz K., Gibula-Bruzda E., Jenda M., Marszalek-Grabska M., Filarowska J., Silberring J., Kotlinska J.H. (2016). Cholinesterase inhibitors, donepezil and rivastigmine, attenuate spatial memory and cognitive flexibility impairment induced by acute ethanol in the Barnes maze task in rats. Naunyn-Schmiedeberg’s Arch. Pharmacol..

[38] Gawel K., Gibula E., Marszalek-Grabska M., Filarowska J., Kotlinska J.H. (2019). Assessment of spatial learning and memory in the Barnes maze task in rodents-methodological consideration. Naunyn-Schmiedeberg’s Arch. Pharmacol..

[39] Uslaner J.M., Parmentier-Batteur S., Flick R.B., Surles N.O., Lam J.S., McNaughton C.H., Jacobson M.A., Hutson P.H. (2009). Dose-dependent effect of CDPPB, the mGluR5 positive allosteric modulator, on recognition memory is associated with GluR1 and CREB phosphorylation in the prefrontal cortex and hippocampus. Neuropharmacology.

[40] Seeber S., Becker K., Rau T., Eschenhagen T., Becker C.M., Herkert M. (2000). Transient expression of NMDA receptor subunit NR2B in the developing rat heart. J. Neurochem..

[41] Calabrese F., Guidotti G., Molteni R., Racagni G., Mancini M., Riva M.A. (2012). Stress-Induced Changes of Hippocampal NMDA Receptors: Modulation by Duloxetine Treatment. PLoS ONE.

[42] Smith P.K., Krohn R.I., Hermanson G.T., Mallia A.K., Gartner F.H., Provenzano M.D., Fujimoto E.K., Goeke N.M., Olson B.J., Klenk D.C. (1985). Measurement of protein using bicinchoninic acid. Anal. Biochem..

[43] Gibula-Tarlowska E., Grochecki P., Silberring J., Kotlinska J.H. (2019). The kisspeptin derivative kissorphin reduces the acquisition, expression, and reinstatement of ethanol-induced conditioned place preference in rats. Alcohol.

[44] Green C.R., Mihic A.M., Nikkel S.M., Stade B.C., Rasmussen C., Munoz D.P., Reynolds J.N. (2009). Executive function deficits in children with fetal alcohol spectrum disorders (FASD) measured using the Cambridge Neuropsychological Tests Automated Battery (CANTAB). J. Child Psychol. Psychiatry.

[45] Spadoni A.D., McGee C.L., Fryer S.L., Riley E.P. (2007). Neuroimaging and fetal alcohol spectrum disorders. Neurosci. Biobehav. Rev..

[46] Popović M., Caballero-Bleda M., Guerri C. (2006). Adult rat’s offspring of alcoholic mothers are impaired on spatial learning and object recognition in the Can test. Behav. Brain Res..

[47] Xu W., Hawkey A.B., Li H., Dai L., Brim H.H., Frank J.A., Luo J., Barron S., Chen G. (2018). Neonatal Ethanol Exposure Causes Behavioral Deficits in Young Mice. Alcohol. Clin. Exp. Res..

[48] Goodlett C.R., Peterson S.D. (1995). Sex differences in vulnerability to developmental spatial learning deficits induced by limited binge alcohol exposure in neonatal rats. Neurobiol. Learn. Mem..

[49] Johnson T.B., Goodlett C.R. (2002). Selective and enduring deficits in spatial learning after limited neonatal binge alcohol exposure in male rats. Alcohol. Clin. Exp. Res..

[50] Wagner J.L., Zhou F.C., Goodlett C.R. (2014). Effects of one- and three-day binge alcohol exposure in neonatal C57BL/6 mice on spatial learning and memory in adolescence and adulthood. Alcohol.

[51] Joshi V., Subbanna S., Shivakumar M., Basavarajappa B.S. (2019). CB1R regulates CDK5 signaling and epigenetically controls Rac1 expression contributing to neurobehavioral abnormalities in mice postnatally exposed to ethanol. Neuropsychopharmacology.

[52] Ieraci A., Herrera D.G. (2020). Early Postnatal Ethanol Exposure in Mice Induces Sex-Dependent Memory Impairment and Reduction of Hippocampal NMDA-R2B Expression in Adulthood. Neuroscience.

[53] Goodfellow M.J., Abdulla K.A., Lindquist D.H. (2016). Neonatal ethanol exposure impairs trace fear conditioning and alters NMDA receptor subunit expression in adult male and female rats. Alcohol. Clin. Exp. Res..

[54] Izquierdo A., Brigman J.L., Radke A.K., Rudebeck P.H., Holmes A. (2017). The neural basis of reversal learning: An updated perspective. Neuroscience.

[55] Bowden S.C., Crews F.T., Bates M.E., Fals-Stewart W., Ambrose M.L. (2001). Neurotoxicity and neurocognitive impairments with alcohol and drug-use disorders: Potential roles in addiction and recovery. Alcohol. Clin. Exp. Res..

[56] Dean A.C., Groman S.M., Morales A.M., London E.D. (2013). An evaluation of the evidence that methamphetamine abuse causes cognitive decline in humans. Neuropsychopharmacology.

[57] Potvin S., Stavro K., Rizkallah E., Pelletier J. (2014). Cocaine and cognition: A systematic quantitative review. J. Addict. Med..

[58] London E.D., Kohno M., Morales A.M., Ballard M.E. (2015). Chronic methamphetamine abuse and corticostriatal deficits revealed by neuroimaging. Brain Res..

[59] Bernheim A., See R.E., Reichel C.M. (2016). Chronic methamphetamine self-administration disrupts cortical control of cognition. Neurosci. Biobehav. Rev..

[60] Wang R., Shen Y.L., Hausknecht K.A., Chang L., Haj-Dahmane S., Vezina P., Shen R.Y. (2019). Prenatal ethanol exposure increases risk of psychostimulant addiction. Behav. Brain Res..

[61] Arias C., Chotro M.G. (2005). Increased preference for ethanol in the infant rat after prenatal ethanol exposure, expressed on intake and taste reactivity tests. Alcohol. Clin. Exp. Res..

[62] Van Waes V., Darnaudéry M., Marrocco J., Gruber S.H., Talavera E., Mairesse J., Van Camp G., Casolla B., Nicoletti F., Mathé A.A. (2011). Impact of early life stress on alcohol consumption and on the short- and long-term responses to alcohol in adolescent female rats. Behav. Brain Res..

[63] Carr G.D., Phillips A.G., Fibiger H.C. (1988). Independence of amphetamine reward from locomotor stimulation demonstrated by conditioned place preference. Psychopharmacology.

[64] Cantacorps L., Montagud-Romero S., Luján M.Á., Valverde O. (2020). Prenatal and postnatal alcohol exposure increases vulnerability to cocaine addiction in adult mice. Br. J. Pharmcol..

[65] Neasta J., Ben Hamida S., Yowell Q., Carnicella S., Ron D. (2010). Role for mammalian target of rapamycin complex 1 signaling in neuroadaptations underlying alcohol-related disorders. Proc. Natl. Acad. Sci. USA.

[66] Everitt B.J., Dickinson A., Robbins T.W. (2001). The neuropsychological basis of addictive behaviour. Brain Res. Brain Res. Rev..

[67] Robinson T.E., Berridge K.C. (2001). Incentive-sensitization and addiction. Addiction.

[68] Weafer J., Gallo D.A., de Wit H. (2016). Effect of Alcohol on Encoding and Consolidation of Memory for Alcohol-Related Images. Alcohol. Clin. Exp. Res..

[69] Toso L., Poggi S.H., Roberson R., Woodard J., Park J., Abebe D., Spong C.Y. (2006). Prevention of alcohol-induced learning deficits in fetal alcohol syndrome mediated through NMDA and GABA receptors. Am. J. Obstet. Gynecol..

[70] Incerti M., Vink J., Roberson R., Wood L., Abebe D., Spong C.Y. (2010). Reversal of alcohol-induced learning deficits in the young adult in a model of fetal alcohol syndrome. Obstet. Gynecol..

[71] Wang N., Chen L., Cheng N., Zhang J., Tian T., Lu W. (2014). Active calcium/calmodulin-dependent protein kinase II (CaMKII) regulates NMDA receptor mediated postischemic long-term potentiation (i-LTP) by promoting the interaction between CaMKII and NMDA receptors in ischemia. Neural Plast..

[72] Shipton O.A., Paulsen O. (2013). GluN2A and GluN2B subunit-containing NMDA receptors in hippocampal plasticity. Philos. Trans. R. Soc. B Biol. Sci..

[73] Ge S., Yang C.H., Hsu K.S., Ming G.L., Song H. (2007). A critical period for enhanced synaptic plasticity in newly generated neurons of the adult brain. Neuron.

[74] Vasuta C., Caunt C., James R., Samadi S., Schibuk E., Kannangara T., Titterness A.K., Christie B.R. (2007). Effects of exercise on NMDA receptor subunit contributions to bidirectional synaptic plasticity in the mouse dentate gyrus. Hippocampus.

[75] Graybiel A.M., Aosaki T., Flaherty A.W., Kimura M. (1994). The basal ganglia and adaptive motor control. Science.

[76] Lovinger D.M. (2010). Neurotransmitter roles in synaptic modulation, plasticity and learning in the dorsal striatum. Neuropharmacology.

[77] Yin H.H., Knowlton B.J. (2006). The role of the basal ganglia in habit formation. Nat. Rev. Neurosci..

[78] Partridge J.G., Tang K.C., Lovinger D.M. (2000). Regional and postnatal heterogeneity of activity-dependent long-term changes in synaptic efficacy in the dorsal striatum. J. Neurophysiol..

[79] Shen W., Flajolet M., Greengard P., Surmeier D.J. (2008). Dichotomous dopaminergic control of striatal synaptic plasticity. Science.

[80] Wang J., Ben Hamida S., Darcq E., Zhu W., Gibb S.L., Lanfranco M.F., Carnicella S., Ron D. (2012). Ethanol-mediated facilitation of AMPA receptor function in the dorsomedial striatum: Implications for alcohol drinking behavior. J. Neurosci..

[81] Sawant O.B., Meng C., Wu G., Washburn S.E. (2020). Prenatal alcohol exposure and maternal glutamine supplementation alter the mTOR signaling pathway in ovine fetal cerebellum and skeletal muscle. Alcohol.

[82] Lee J., Lunde-Young E.R., Naik V., Ramirez J., Orzabal M., Ramadoss J. (2020). Chronic Binge Alcohol Exposure During Pregnancy Alters mTOR System in Rat Fetal Hippocampus. Alcohol. Clin. Exp. Res..

[83] Morisot N., Phamluong K., Ehinger Y., Berger A.L., Moffat J.J., Ron D. (2019). mTORC1 in the orbitofrontal cortex promotes habitual alcohol seeking. eLife.

